# Broken Needle Embedded in the Body during Vascular Puncture

**DOI:** 10.3390/healthcare10081436

**Published:** 2022-07-31

**Authors:** Hye Sook Choi

**Affiliations:** Department of Internal Medicine, Kyung Hee University Medical Center, #23 Kyungheedae-ro, Dongdaemun-gu, Seoul 02447, Korea; maxymus72@hanmail.net

**Keywords:** broken needle, vascular puncture, fluoroscopic radiography

## Abstract

The use of needles is essential in most medical procedures and surgery; however, needle breakage is not known to happen very frequently. Even if it does, it is most likely to occur during dental procedures, sutures, aspiration, biopsy, anesthesia, and drug abuse. To our knowledge, this is the first report about needle detachment from the syringe during a vascular puncture for blood collection. In this case, an 87-year-old obese woman with generalized edema had repeated vascular punctures to the femoral artery for arterial blood gas analysis (ABGA). After blood collection at this instance, when the syringe was pulled out, the needle was detached from it. Radiography revealed that the broken needle was lodged in the groin. A surgical incision with the fluoroscopic radiography located the embedded needle in the soft tissue and allowed the retrieval of its fragments from the groin soft tissue. Obesity and repeated punctures may increase the risk of needle breakage and prior inspection of needles for such procedures may be necessary.

## 1. Introduction

With the development of medicine, the practice for treatment or diagnosis is also increasing. Most essential medical procedures use needles; for instance, procedures for drug injections, biopsy, aspiration, suture, anesthesia, or acupuncture require the use of needles. Even people who inject drugs use their own needles. As the number of procedures using needles increases, the number of needle-related problems also increase. Limited visibility, difficult access, sudden movement of patients, inappropriate size of the instruments, lack of experience of operators or mishandling by them [1], and hard site of injection may increase the risk of needle-related problems such as needle breakage, displacement, embedding, or residue [1]. The above-mentioned risk factors, leading to increased instances of needle breakage are related to the operator or patient. Another risk factor to consider as the cause of needle breakage is a defect in the needle itself. The premise is that the needle used for medical purposes should not be defective. Here, we report a case of needle breakage, by the way of separation from the syringe, during the process of vascular puncture, along with the relevant literature review. 

## 2. Case Report

**Figure 1:**
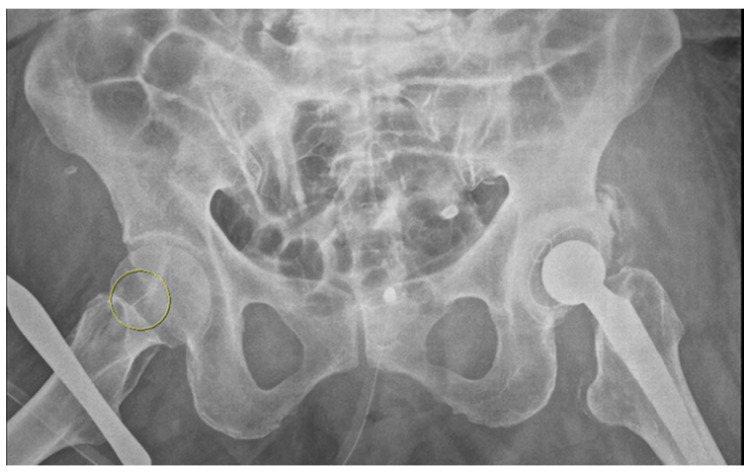
Radiography of pelvic bone. A needle fragment showed in the right groin (yellow circle).

**Figure 2:**
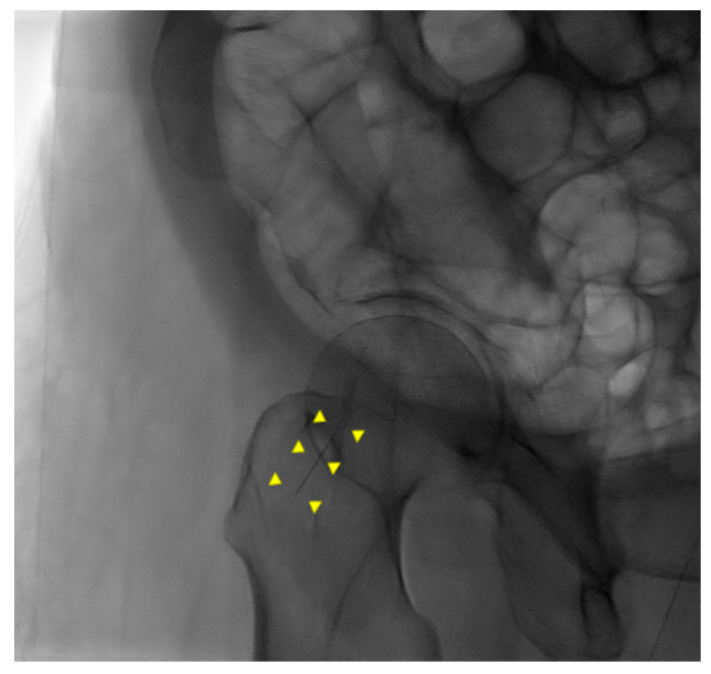
Fluoroscopic radiography of pelvic bone. A needle fragment showed in the right groin (yellow triangles).

**Figure 3:**
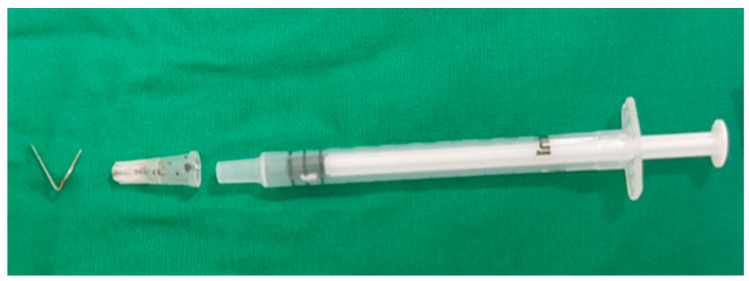
A piece of needle fragment retrieved from the groin and the used syringe. The bent needle while pulling it out with mosquito forceps.

An 87-year-old female patient came to the emergency department with increased bilateral pleural effusion with pneumonia, and she was admitted for treatment. She had been bedridden with comorbidities, including old cerebral infarction, hypertension, and had left femur neck fracture with endoprosthesis for 4 weeks before admission. Her body mass index was 26.67 kg/m^2^. She was generally edematous. We drained pleural effusion, diagnosed as parapneumonic effusion, and administered intravenous antibiotics for the treatment of pneumonia with parapneumonic effusion. During hospitalization, her conditions were wax and wane, with aspiration pneumonia. On day 37 of hospitalization, her blood pressure was consistently low despite the use of a vasopressor. For arterial blood gas analysis (ABGA) to monitor the patient’s state of acidosis, we repeatedly performed puncture of the right femoral artery instead of the radial or dorsal artery. We used a 25 gauge and 16 mm length needle for femoral arterial puncture. After blood extraction, while drawing the syringe out of a femoral artery, the needle was disconnected from the syringe. The needle remained in the groin of the patient, and its location was not identified through portable ultrasonography. Radiography of the pelvic bone showed the needle fragment in the right groin (Figure 1). Fluoroscopic radiography revealed the exact location of the needle fragment (Figure 2). A surgical incision was performed to obtain the embedded needle in the soft tissue of the groin, without penetrating the femoral artery. The needle fragment was removed (Figure 3) completely without complications. The needle was bent as it was pulled out with mosquito forceps (Figure 3).

## 3. Discussion

The incidence of a broken needle or needle breakage is not unknown. Broken needles have been reported during dental procedures [2,3], laparoscopy [4], surgical operation [1], percutaneous needle biopsy [5], endobronchial ultrasound-guided-transbronchial needle aspiration [6], antegrade suture passer of shoulder [7], bone biopsy [8], acupunctures [9], spinal anesthesia [10,11], and the injection of drugs [12,13]. Seven case reports of broken needles during various procedures were reported in 2021 from a PubMed^®^ search. However, there have been no reports about needle breakage during blood puncture for blood collection. In this current case, we collected blood from the femoral artery for a very short time, using a 16mm needle; however, the needle was embedded in the groin tissue while the syringe was withdrawn. To our knowledge, this is the first report of needle breakage during arterial puncture for ABGA. 

In this case, while pulling out the syringe, the needle separated from the syringe and remained embedded in the tissue. The potential reasons for needle detachment could be syringe defects, fault in handling by the operator, or the patient. Risk factors associated with needle breakage of antegrade suture passer are thicker tendon, higher tendinosis, and delaminated tears [7]. Saurin et al. reported that emergency procedures, morbid obesity and multiple puncture attempts, redirection without introducer, poorly differentiated landmarks, and resistance to needle advancement increase the risk of a broken needle during spinal anesthesia [11]. In our case, the patient was obese and had repeated punctures for ABGAs into the femoral artery, which could be considered as the cause of needle breakage. Additionally, the insertion of a very thin needle into the femoral artery might also be considered as the cause of needle detachment from the syringe. The thin needle was obstructed from being pulled out along with the syringe because repeated punctures, in addition to obesity, had increased the tissue resistance. We could not exclude the defective connection between the syringe and the needle. Operators usually do not check for defects in the needle because it is generally believed that flawless medical instruments would be delivered. We suggest that factors such as appropriate needle size for the vessel, proper location to be punctured, and prior identification of syringe defects should be considered for vascular puncture. 

Sometimes, needle fragments could migrate to a remote site via blood flow [13] and penetrate blood vessels [5,14] or soft tissues [2,13]. This could be life-threatening. Thus, the need for prompt retrieval of broken needle fragments is important. In this case, fortunately, the needle fragment did not remain in the femoral artery but was retained by the soft tissue. 

Sometimes it may not be possible to locate retained needle fragments if they become embedded in the tissue or move to some other site during the procedure. Therefore, depending on the direction and depth of the needle fragments remaining in the tissue, ultrasonography may not always be able to identify the needle fragments [12]. In our patient’s case, ultrasonography could not discover the needle fragment in the groin; it was revealed using radiography. 

If broken needle fragments during surgery remain free at the surgical site, without becoming embedded, it is helpful to use a strong magnet in a sterile transparent sheath. Needle fragments stick to the magnet and can be seen and removed easily [15]. Titus at al. reported a broken suture needle during a laparoscopic cholecystectomy, with the free shard becoming lodged into the surrounding adipose tissue [4]. It was identified by intraoperative C-arm radiography and was removed using a pacemaker magnet [4]. However, a broken needle embedded completely in the soft tissue can be removed by surgical exploration [1,13,14]. A broken needle embedded in the bone can be retrieved using a drill bit [8,16]. Nikhil et al. reported that a broken needle fragment during an endobronchial ultrasound-guided transbronchial needle aspiration procedure, was removed using alligator forceps [6]. Broken acupuncture needles can be removed by pulling out, surgical exploration, laparoscopy, laparotomy, thoracotomy, or video-associated thoracoscopy [9]. In people who inject drugs, needle fragments might not be recognized until long after being broken, and therefore tend to remain in the body for a longer amount of time [12,13]. In some cases, the needle fragment that remains in the body cannot be removed due to patient refusal [12]. Fluoroscopic radiography can help in removing needles successfully without complications [5]. Our patient underwent surgery to remove the needle fragment under fluoroscopic radiography, and it was removed completely without any complications. The patient has not sued the hospital or the doctors to obtain compensation for the damage.

## 4. Conclusions

Needles may break and remain in the body during surgery, procedure, or injection, or even a simple vascular puncture for blood collection. It is important that the broken needle is located and removed quickly and carefully. For vascular puncture for blood collection, the selection of proper needle size and appropriate puncture site, with advance inspection of the needle, may be necessary to avoid breakage. 

## Data Availability

Not applicable.
